# Apical periodontitis and glycemic control in type 2 diabetic patients: Cross-sectional study

**DOI:** 10.4317/jced.57191

**Published:** 2020-10-01

**Authors:** Flor de Liz Pérez-Losada, José López-López, Jenifer Martín-González, Enric Jané-Salas, Juan J. Segura-Egea, Albert Estrugo-Devesa

**Affiliations:** 1 DDS, Doctoral fellow, Department of Odontostomatology, Faculty of Medicine and Health Sciences, University of Barcelona. L’Hospitalet de Llobregat, Barcelona, Spain; 2 MD, DDS, PhD, Professor, Department of Odontostomatology, Faculty of Medicine and Health Sciences, University of Barcelona- Dental Hospital, University of Barcelona, L’Hospitalet de Llobregat, Barcelona, Spain; 3 DDS, PhD, Associate Professor, Division of Endodontics, Department of Stomatology, School of Dentistry, University of Sevilla, Sevilla, Spain; 4 MD, DDS, PhD, Professor, Department of Odontostomatology, Faculty of Medicine and Health Sciences, University of Barcelona- Dental Hospital, University of Barcelona, L’Hospitalet de Llobregat, Barcelona, Spain; 5 MD, DDS, PhD, Professor, Division of Endodontics, Department of Stomatology, School of Dentistry, University of Sevilla, Sevilla, Spain; 6 MD, DDS, PhD, Associate Professor, Department of Odontostomatology, Faculty of Medicine and Health Sciences, University of Barcelona- Dental Hospital, University of Barcelona, L’Hospitalet de Llobregat, Barcelona, Spain

## Abstract

**Background:**

The objective of this study was to analyze the possible relationship between the glycemic control and the prevalence of apical periodontitis in type 2 diabetic patients. The null hypothesis was that apical periodontitis is not associated with glycemic control.

**Material and Methods:**

In a cross-sectional design, the radiographic records of 216 type 2 diabetic patients (65.0 ± 10.7 years), 117 men (54.2%) and women (45.8%), were examined. Glycated hemoglobin (HbA1c) was used to assess glycemic control, considering an HbA1c level < 6.5% as well-controlled diabetes. Apical periodontitis was diagnosed as radiolucent periapical lesions using the periapical index score. The Student t test, chi-square test, and logistic regression analysis were used in the statistical analysis.

**Results:**

The average HbA1c value was 7.0 ± 2.2%. Forty seven (21.8%) had HbA1c levels under 6.5% (mean ± SD = 6.0 ± 2.2%), being considered well-controlled patients, and 169 (78.2%) had an HbA1c level ≥ 6.5% (mean ± SD = 7.8 ± 2.24%), being considered poor controlled patients. Forty four per cent of diabetics had apical periodontitis, 12.5% had root-filled teeth, and 52.3% had root filled teeth with radiolucent periapical lesions. No significant differences were observed in any of these three variables between patients with good or poor glycemic control. In the multivariate logistic regression analysis the presence of radiolucent periapical lesions in at least one tooth did not correlate significantly with HbA1c levels (OR = 1.4; 95% C.I. = 0.70 – 3.09; *p* = 0.31).

**Conclusions:**

The results reveal no association of glycemic control with the prevalence of apical periodontitis or root canal treatment in diabetic patients.

** Key words:**Apical periodontitis, diabetes mellitus, endodontic medicine, glycated haemoglobin.

## Introduction

Diabetes mellitus (DM) is a group of metabolic diseases affecting the metabolism of carbohydrates, lipids and proteins, with hyperglycemia, as a result of a deficiency in insulin secretion, lack of insulin action or both (1). Chronic hyperglycemia is associated with long standing damage, dysfunction and failure of diverse organs, especially eyes, kidneys, nerves, heart and blood vessels (2). Glycated hemoglobin (HbA1c) has been used as the gold standard for the control of diabetic patients. This test measures the average glycaemia of the last 2-3 months, allowing the assessment of the effectiveness of the patient’s treatment (3). The American Association of Clinical Endocrinologist considers HbA1c ≥ 6.5% as a goal in the optimal control of diabetic patients (4).

Apical periodontitis (AP) is an inflammatory lesion around the root apex consecutive to bacterial infection of the pulp canal system (5). A periapical radiolucent lesion (PRL) in the radiography is the characteristic sign of chronic apical periodontitis (6). However, AP must be considered not exclusively a locally process. The inflammatory cytokines released in inflamed periapical tissues, such as IL-1β, IL-6, IL-8, and TNF-α, may reach the systemic circulation inducing or perpetuating an elevated chronic systemic inflammatory status (7-9).

Several studies have reported results supporting a relationship between the prevalence AP and diabetes (10-17). Moreover, several systematic reviews and meta-analyses have found significant association between the outcome of endodontic treatment and diabetes (18-20). Nevertheless, few studies have investigated the possible relationship between endodontic infections and glycemic control in diabetic patients. Poorly controlled diabetics tend to develop periapical radiolucencies during endodontic treatment (21), and chronic apical periodontitis has been linked to an increase in blood glucose in diabetic patients (22). Two studies have found correlation between higher prevalence of AP and poor glycemic control in diabetic patients (17,23). Vice versa, it has been found a higher percentage of reduction of periradicular radiolucencies in patients with lower glycaemia (24). However, there are no definitive data on the association between blood glucose levels and the prevalence of AP in diabetic patients.

The aim of this study was to analyze the possible relationship between the prevalence of AP, diagnosed as a periapical radiolucent lesion (PRL), and the glycemic control, assessed by HbA1c levels, of type 2 diabetic patients. The null hypothesis was that AP is not associated with glycemic control in diabetic patients.

## Material and Methods

An observational descriptive cross-sectional study was designed. The study was approved by the ethical committee of the Dental Clinic of the University of Barcelona (Barcelona, Spain), reference number 10/31/2018. The investigation was conducted following the World Medical Association-Declaration of Helsinki. All participants signed the written informed consent.

-Patients

Participants were recruited among patients presenting consecutively seeking routine dental care (not emergency care) at the Dental Hospital (Faculty of Medicine and Health Sciences, Dentistry, University of Barcelona, HOUB) and from a primary health center (ABS Sta. Eulalia Sud, L’Hospitalet de Llobregat, Barcelona), during the years 2013 through 2016. All type 2 diabetic patients, diagnosed according to the current criteria for the diagnosis of diabetes (25), were asked to voluntarily participate. The following inclusion criteria were used: over 18 years of age, with more than seven teeth remaining, with records of HbA1c levels in the last week, who accepted a radiological examination. The exclusion criteria were the following: less than 18 years of age, less than eight remaining teeth, unknown HbA1c levels, or no acceptance of the radiological examination. Two hundred and forty four diabetic patients met the inclusion/exclusion criteria and were asked to participate in the study, but only 216 (65.0 ± 10.7 years), 117 men (54.2%) and women (45.8%) accepted to be included in the study and signed the written informed consent.

Data was collected from the clinical histories, eliciting information on medical and dental history, about coronary heart disease, the most recent measurement of HbA1c levels, smoking status, alcohol consumption assessed as intake of Standard Beverage Unit (8-13 grs of pure alcohol) by day, and periodontal status assessed using the criteria of Machtei *et al.* (26).

-Assessment of glycemic control 

The status of the metabolic control of the diabetic patients was achieved by checking the values of the glycosylated hemoglobin (HbA1c). The blood test had to be performed less than a month prior to the study visit. Adequate glycemic control was defined as HbA1c < 6.5% (25).

-Radiological examination and periapical status assessment

Radiographic periapical status was diagnosed on the basis of examination of digital panoramic radiographs of the jaws. Two qualified radiologic technicians, with more than 10 years of experience in their field, performed the OPG using a digital orthopantomogram (Promax, Planmeca, class 1, type B, 80 KHz; Planmeca, Helsinki, Finland).

All teeth, excluding third molars, were recorded. Teeth were categorized as root filled teeth if they had been filled with a radiopaque material in the root canal(s). For each subject the number of teeth present, the number and location of root-filled teeth, and the number and location of teeth having identifiable radiolucent periapical lesions were recorded.

The periapical status was assessed using ‘Periapical Index’ (PAI) score (27). A score greater than 2 (PAI ≥ 3) was considered to be a sign of AP. The worst score of all roots was taken to represent the PAI score for multi-rooted teeth.

-Observers’ calibration

Three observers with a broad clinical experience in endodontics assessed the radiographs (AED, JLL and FPL); disagreements were weighted by JSE and JMG. Before evaluation, the observers participated in a calibration course for PAI system, which consisted of 100 radiographic images of teeth, some root-filled and some not, kindly provided by Dr. Ørstavik (Ørstvik *et al.* 1986). Each tooth was assigned to one of the PAI scores by using visual references (also provided by Dr. Ørstavik) for the 5 categories within the scale. After scoring the teeth, the results were compared to a “gold standard atlas”, and a Cohen Kappa was calculated (0.78 – 0.89).

Intra-observer reproducibility was evaluated for each examiner. Every observer scored the panoramic radiographs of 20 patients (10 of each group, randomly selected). Then, one month after this first examination, the observer was recalibrated in the PAI system and repeated the scoring of the radiographs of the same 20 patients. The intra-observer agreement test on PAI scores on the 20 patients produced a Cohen’s Kappa ranging 0.80 - 0.91.

Intra-observers reproducibility was also determined comparing the PAI scores on the 20 radiographs provided by each observer. The agreement test produced a Cohen’s Kappa ranging 0.78 - 0.94. The Cohen’s Kappa for inter-observers variability ranged 0.83 - 0.90. The consensus radiographic standard was the simultaneous interpretation by the three examiners of the panoramic radiograph of each patient.

-Statistical analysis

Minimal sample size was calculated using “Sample size and power calculator”, v7.12 software (https://www.imim.cat/ofertadeserveis/software-public/granmo/). Accepting an alpha risk of 0.05 and a beta risk of 0.2 in a two-sided test, 204 subjects are necessary in the observed group to recognize a difference greater than or equal to 0.1 units. A proportion in the reference group has been estimated to be 0.35. It has been anticipated a drop-out rate of 10%. Post-hoc power analysis has been performed using the same software.

The primary data was introduced into an Excel spreadsheet (Microsoft Corp, Redmond, WA). SPSS (version 11; SPSS, Inc., Chicago, IL) was used for the statistical analysis. Data is presented as media ± standard deviation. A t-test and X2 test were used to determine the differences between groups. A logistic regression analysis was performed to measure the strength of the association between the HbA1c levels and the presence of periapical radiolucencies, adjusting the presence of co-variables. A *p* value <0.05 was considered to be statistically significant.

## Results

The characteristics and dental status of the type 2 diabetic patients are described in Table 1. The mean number of teeth was 20.9 ± 6.6, and the average number of teeth with radiolucent periapical lesion was 0.5 ± 0.4. The mean number of RFT per patient was 0.8 ± 1.3. Sixty patients (27.8%) were smoker or former smoker, 52 (24.1%) were consumers of alcoholic beverages, 150 (69.4%) had coronary heart disease, and 130 (60.2%) had periodontal disease. The average glycosylated hemoglobin (HbA1c) value was 7.0 ± 2.2 per cent.

**Table 1:**
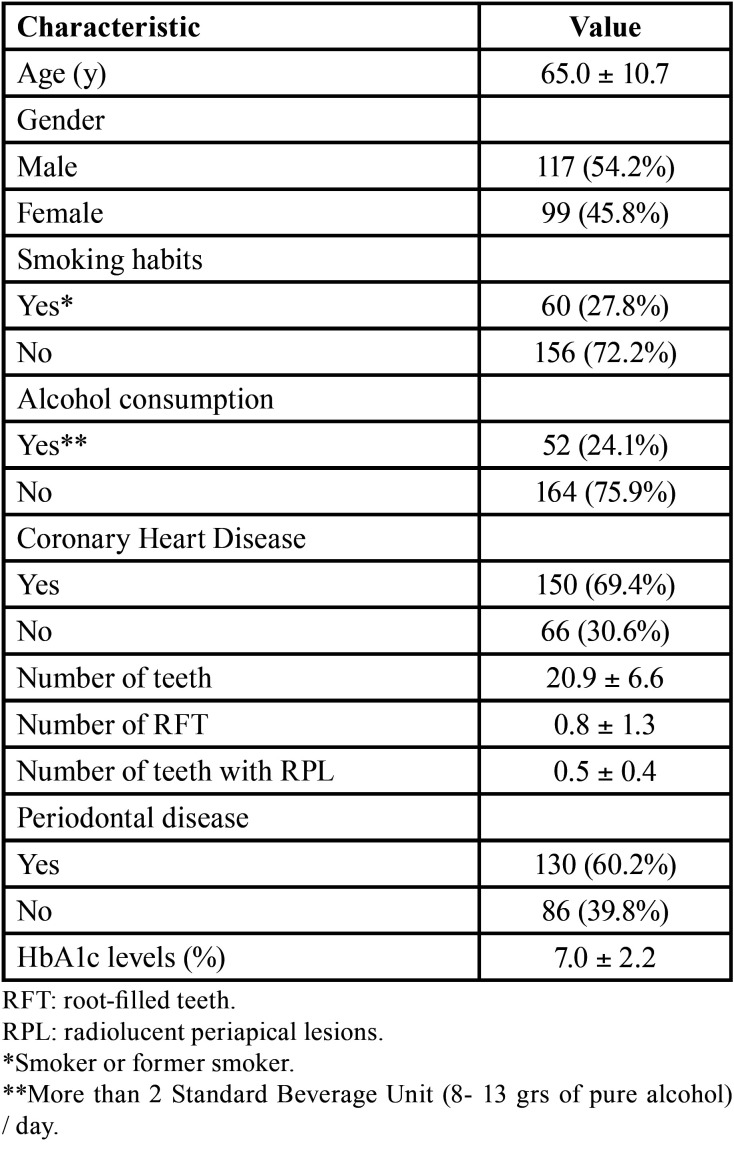
Characteristics and dental status of diabetic patients (n = 216) included in the study. Results are expressed as mean ± standard deviation.

Diabetic patients were classified dichotomously according to their glycemic control (Table 2). Forty seven (21.8%) had HbA1c levels under 6.5% (mean ± SD = 6.0 ± 2.2%), being considered well-controlled patients. On the contrary, 169 (78.2%) had an HbA1c level ≥ 6.5% (mean ± SD = 7.8 ± 2.24%), being considered poor controlled patients. No significant differences were found between patients with good or poor glycemic control regarding age, gender, number of teeth, smoking habits, alcohol consumption, coronary heart disease or periodontal status (*p* > 0.05). Forty four per cent of diabetic patients showed at least 1 radiolucent periapical lesion (RPL), 12.5% had at least 1 RFT, and 52.3% had at least 1 root-filled tooth with RPL (Table 3). There was no significant difference between patients with good or poor glycemic control in the number of teeth with periapical lesions (OR = 1.74; 95% C.I. = 0.89 – 3.42; *p* = 0.10). The percentage of good-controlled patients with at least 1 root-filled tooth with RPL was 34.0%, whereas amongst poor-controlled patients this percentage was 47.3% (*p* > 0.05). The number of RFT (OR = 1.26; 95% C.I. = 0.45 – 3.52; *p* = 0.66), and the number of RFT with periapical lesions (OR = 1.33; 95% C.I. = 0.69 – 2.53; *p* = 0.39) was not associated to HbA1c levesl.

**Table 2:**
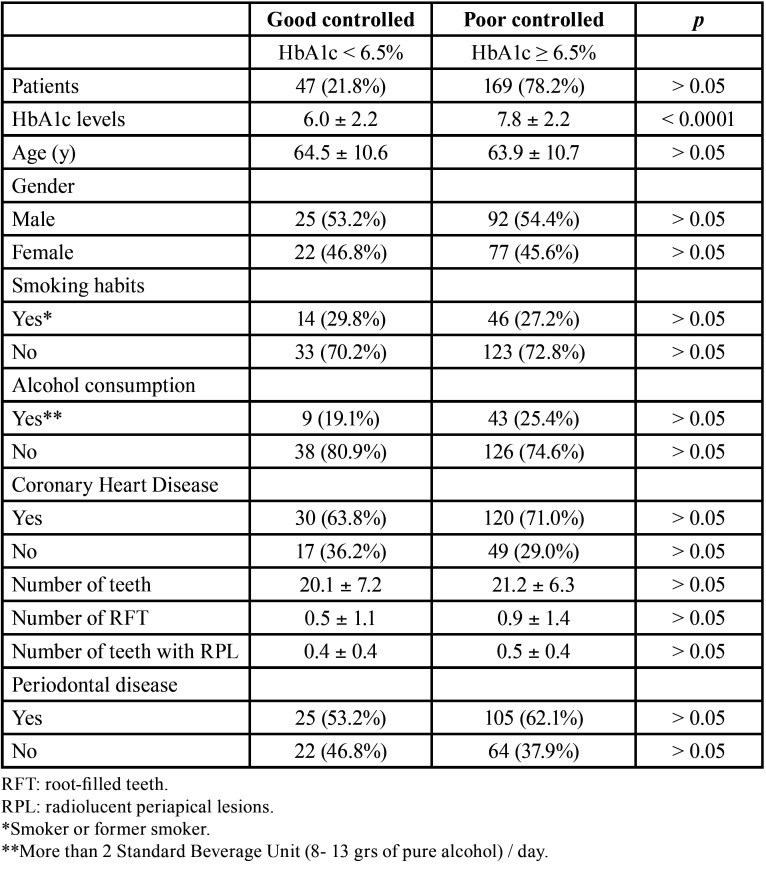
Characteristics and dental status of diabetic patients after classifying them according to their glycemic control (HbA1c levels). Results are expressed as mean ± standard deviation.

**Table 3:**
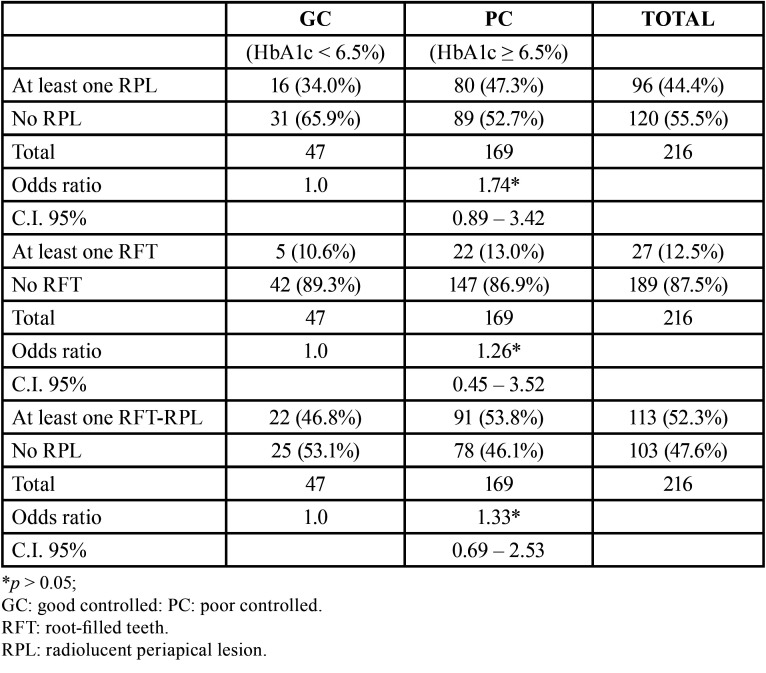
Univariate logistic regression analysis of the association of the independent variables a) presence of radiolucent periapical lesions (RPL), b) root filled teeth (RFT), and c) RFT with RPL (RFT-RPL), with the dependent variable “HbA1c levels “, dichotomized as ˂ 6.5% (GC) or ≥ 6.5% (PC).

To further investigate whether HbA1c levels were related to endodontic variables, multivariate logistic regressions were run with age, gender, smoking habits, alcohol consumption, number of teeth, number of RFT, number of RFT with AP, periodontal status, and periapical status as independent explanatory variables, and HbA1c levels as dependent variable (0 = HbA1c ˂ 6.5%; 1 = HbA1c ≥ 6.5%) (Table 4). In the multivariate logistic regression analysis including all the above factors as covariates, the presence of RPL in at least one tooth did not correlate significantly with HbA1c levels (OR = 1.4; 95% C.I. = 0.70 – 3.09; *p* = 0.31).

**Table 4:**
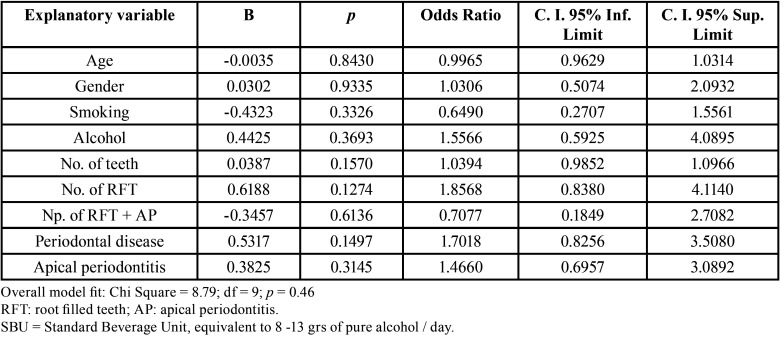
Multivariate logistic regression analyse of the influence of the independent variables age, gender (0 = women, 1 = male), smoking (0 = non-smoker, 1 = smoker), alcohol consumption (0 = < 2 SBU, 1 = > 2 SBU), number of teeth, number of RFT, number of RFT with AP, periodontal disease (0 = absent, 1 = present), and apical periodontitis (0 = no tooth with radiolucent periapical lesion, 1 = one or more tooth with radiolucent periapical lesion), on the dependent variable “HbA1c levels” (0 = HbA1c < 6.5%; 1 = HbA1c ≥ 6.5%).

## Discussion

In this observational cross-sectional study, the possible association between the prevalence of RPL and glycemic control in diabetic patients has been investigated. The results reveal no higher prevalence of RPL in poor controlled type 2 diabetic patients, with HbA1c levels ≥ 6.5% (*p* = 0.31). Nor has an association been found between the level of HbA1c and the prevalence of RCT.

The recruitment method of the patients was similar to that used in previous studies (10,14,23,28): subjects presenting consecutively seeking routine dental care (not emergency care) at the dental service of the Faculty of Dentistry. Diabetes was diagnosed according to the current criteria for the diagnosis of diabetes (25). Glycated hemoglobin levels (HbA1c) were used to assess glycemic control, providing an accurate measure of blood glucose levels in the previous 30-90 days (4). When blood glucose levels are high, glucose and other sugars slowly bind covalently and non-enzymatically to hemoglobin (glycosylation). The rate of formation of HbA1c is directly proportional to blood glucose concentrations.

The prevalence of AP was assesses using panoramic radiographs and the Periapical Index (27), as other studies have used previously (29-35). Observer calibration was performed according to PAI score system, obtaining adequate Kappa values for intra-observer and inter-observer reproducibility (27). Panoramic radiographs showed high specificity and positive predictive value diagnosing apical periodontitis (36), can be obtained with convenience and speed, and have the advantage of producing an average patient exposure of only 4.1μSv (37).

In the present study, the percentage of diabetics with good glycemic control was very low. Only 47 diabetic patients (22%) had well-controlled HbA1c levels (< 6.5%). Sanchez-Domínguez *et al.* (23) found a similar proportion (28.9%), but other studies have reported higher percentages of well-controlled diabetics (17,40). This lower percentage of well-controlled diabetics could explain, at least in part, the differences in the results of the present study with those previously performed (17,23,40). It may also have influenced the non-significant findings of the present study, since the high percentage of poorly controlled diabetic patients limits the statistical power to detect significant differences between the proportions of RPL, RFT and RFT with RPL in both groups. Before starting this study, the researchers calculated that they would need a minimal sample size of 204 participants to see a statistically significant difference between poor- and good-controlled diabetic patients. The high percentage of poor-controlled diabetics may also have caused that age, gender, smoking habits, alcohol consumption, coronary heart disease, number of teeth, number of root-filled teeth, or number of teeth with RPLs did not show statistical association with HbA1c levels (*p* > 0.05). However, other studies have also found no association between these variables and glycemic control in diabetic patients (23).

The present results show that 44.4% of diabetic patients had AP, diagnosed as RPL, in one or more teeth. Some previous studies have found higher prevalence of periapical lesions in diabetics (10,28,38), but other have reported minor prevalence values (14,17,39). The prevalence of at least one RPL in well-controlled diabetics did not significantly differ from that observed in poor-controlled diabetic patients (OR = 1.74; *p* = 0.10). The high percentage of poorly controlled diabetics have decreased the power of the study. Accepting an alpha risk of 0.05 in a two-sided test with 169 poor-controlled patients and 47 good-controlled diabetics, the statistical power is only 37.4% to recognize as statistically significant the difference from 47% of prevalence of RPL in the first group to 34% in the second group.

Only three epidemiological studies have investigated the potential association of periapical inflammation with the glycemic control in diabetics, two using digital panoramic radiographs and the PAI system score (17,23), and another (40) using CBCT with a voxel size of 0.200 mm and the cone beam computed tomography periapical index (CBCTPAI) (41). The study conducted in 2015 included 83 diabetic patients and used the same HbA1c threshold (6.5%) to determine the good or poor glycemic control, reporting an OR = 3.6 (95% C.I = 1.0 – 13.0; *p* = 0.049), calculated through multivariate logistic regression analysis, concluding that HbA1c levels of diabetic patients are associated with periapical status (23). Smadi (17), including 145 diabetic patients and using a 7% HbA1c level to establish the degree of glycemic control, found that poor controlled diabetics showed higher prevalence of AP lesions (18.3%) compared with well-controlled diabetic patients (9.21%) (*p* = 0.001). Moreover, poor controlled diabetic showed higher prevalence of RFT with AP (32.0%) compared to well-controlled patients (21.8%) (*p* = 0.02), concluding that a poor glycemic control may be associated with a higher prevalence of AP and increased rate of endodontic failures. The third study analysed only the periapical status of RFT (40), including a small sample (43 diabetic patients) and assessing the periapical status using the CBCTPAI index (41). In this study the HbA1c level to assess glycemic control was 6.5%, finding 69.6% of well-controlled diabetics, and concluding that there were no differences in the periapical status of RFT between well- and poor-controlled diabetics. The characteristics of the samples, the different threshold of HbA1c values, the different proportions of well- and poor-controlled diabetics, and the radiological technique and criteria used to diagnose periapical lesions may explain the observed differences amongst these studies and the present results.

In accordance with the study of Sisli (40), the results of the present study did not find association between root canal treatment (RCT) outcome and glycemic control. The prevalence of at least 1 RFT with RPL was similar in well- and poor-controlled diabetic patients (*p* > 0.05). However, the total prevalence of RPL associated to endodontically treated teeth was high (52.3%). Other previous studies have found similar percentages (10,23,28). Scientific evidence support the relationship between poor RCT outcome and diabetes (18). Two systematic reviews with meta-analysis have shown that diabetics are more likely to have periapical radiolucent lesions in their RFT (OR=1.4; 95% CI = 1.1 to 1.8; *p* = 0.006) (20) and that they are more likely to lose RFT (OR = 2.4; 95% CI = 1.5–3.9; *p* = 0.0001) (19).

The possible relationship between glycemic control and endodontic infection has been also investigated in animal models. It has been demonstrated that diabetes enhance the development of periradicular lesions in rats (42), and hyperglycaemia adversely affects pulp healing in rats after pulp capping with MTA (43). Moreover, HbA1c in diabetic rats is increased by oral infections (43).

The results of these animal studies, together with those of human epidemiological studies (17,23,40) suggest the existence of an association between glycemic control and endodontic infection. The mismatch of the results of the present study may be due to the high proportion of diabetic patients with poor glycemic control (78%). In fact, the proportion of patients with at least one lesion of AP among poorly controlled diabetics was 47%, while in well-controlled patients, it was 34%, 13 points lower, but the difference was not statistically significant probably because of the high percentage of poor-controlled diabetics. A case-control design, including the same number of diabetics well- and poor-controlled, could provide more consistent data.

## Conclusions

The results reveal no association of glycemic control with the prevalence of apical periodontitis or root canal treatment in diabetic patients.
